# Global Longitudinal Strain as a Prognostic Biomarker for Asymptomatic Moderate to Severe Aortic Regurgitation with Preserved Ejection Fraction: A Systematic Review and Meta-Analysis

**DOI:** 10.3390/jcm14186534

**Published:** 2025-09-17

**Authors:** Myung-Rho Kim, Taha Shaikh, Spencer Taylor, Shawn Wang, Darren Nguyen, Banveet Kaur Khetarpal, Ali Namazi, Vidhani S. Goel, Roberto Sagaribay, Kavita Batra

**Affiliations:** 1 Department of Internal Medicine, Kirk Kerkorian School of Medicine at UNLV, University of Nevada, Las Vegas, Las Vegas, NV 89106, USA; taha.shaikh@unlv.edu (T.S.); darren.nguyen@unlv.edu (D.N.); 2 Kirk Kerkorian School of Medicine at UNLV, University of Nevada, Las Vegas, Las Vegas, NV 89106, USA; taylos21@unlv.nevada.edu (S.T.); wangs23@unlv.nevada.edu (S.W.); 3 Division of Cardiovascular Medicine, Department of Internal Medicine, Kirk Kerkorian School of Medicine at UNLV, University of Nevada, Las Vegas, Las Vegas, NV 89106, USA; banveet.kaur@unlv.edu (B.K.K.); ali.namazi@unlv.edu (A.N.); 4 School of Public Health, University of Nevada, Las Vegas, Las Vegas, NV 89154, USA; vidhani.goel@unlv.edu; 5 Office of Research Kirk Kerkorian School of Medicine at UNLV, University of Nevada, Las Vegas, Las Vegas, NV 89102, USA; roberto.sagaribay@unlv.edu; 6 Department of Medical Education, Kirk Kerkorian School of Medicine at UNLV, University of Nevada, Las Vegas, Las Vegas, NV 89106, USA

**Keywords:** global longitudinal strain (GLS), aortic regurgitation (AR), preserved ejection fraction, prognostic biomarkers, risk stratification, asymptomatic severe AR, meta-analysis, systematic review, left ventricular ejection fraction (LVEF), cost-effectiveness

## Abstract

**Background/Objectives**: Chronic aortic regurgitation (AR) can remain asymptomatic despite progressive myocardial damage. While current surgical guidelines rely heavily on left ventricular ejection fraction (LVEF) and symptoms, these measures may fail to detect subclinical myocardial dysfunction. Global longitudinal strain (GLS), a sensitive echocardiographic marker, has emerged as a potential early biomarker in valvular disease. Therefore, this systematic review aims to assess whether impaired GLS is significantly associated with adverse clinical outcomes including symptom onset, reduced LVEF, and the need for aortic valve intervention among patients with asymptomatic moderate to severe AR and preserved LVEF. **Methods**: This systematic review and meta-analysis followed PRISMA guidelines and was registered with PROSPERO (CRD42024579540). Comprehensive searches of PubMed, Embase, and the Cochrane Library were completed on November 28, 2024. Screening, deduplication, and blinding were conducted using Rayyan. Eligible studies included adults with chronic, asymptomatic moderate to severe AR and preserved LVEF who underwent GLS measurement. Risk of bias was assessed using the National Heart, Lung, and Blood Institute (NHLBI) tool. A random-effects meta-analysis was performed on studies reporting multivariate hazard ratios (HRs) and 95% confidence intervals. **Results:** Twelve studies (N = 3278) were included in the systematic review, and four studies (n = 1125) were eligible for meta-analysis. Impaired GLS was significantly associated with adverse outcomes, yielding a pooled HR of 1.359 (95% CI: 1.106–1.668; *p* = 0.003). Heterogeneity was substantial (I^2^ = 77%), and the 95% prediction interval ranged from 0.553 to 3.340, indicating potential variability in future settings. **Conclusions**: GLS is a promising prognostic biomarker for identifying subclinical cardiac dysfunction in asymptomatic moderate to severe AR with preserved LVEF. Its use may enhance early risk stratification and optimize timing for surgical intervention. Larger prospective studies are needed to validate specific GLS thresholds for clinical decision-making.

## 1. Introduction

Aortic regurgitation (AR) is the third most common valvular heart disease, with moderate to severe AR affecting approximately 4.5% of the elderly population [1]. Clinical manifestations of AR range from shortness of breath and chest pain to sudden cardiac death, necessitating surgical intervention [1,2]. However, chronic moderate to severe AR can remain asymptomatic for extended periods. In such cases, there is a Class I recommendation for surgical intervention if left ventricular ejection fraction (LVEF) is reduced, due to the high mortality associated with untreated AR [1,2,3,4]. When LVEF is preserved, there is a Class IIa recommendation for surgery, but only if there is severe left ventricular (LV) enlargement, which is defined as a left ventricular end-systolic diameter (LVESD) greater than 50 mm or a left ventricular end-diastolic diameter (LVEDD) greater than 65 mm [4,5]. Close monitoring with routine echocardiography is essential during the asymptomatic phase to detect LV dysfunction and changes in LV dimensions [6,7].

Chronic AR may initially remain asymptomatic due to compensatory remodeling of the LV. Over time, however, increasing wall tension leads to LV dilation and hypertrophy to accommodate elevated end-diastolic volume and maintain high cardiac output [8]. Eventually, the LV can no longer compensate for the volume caused by blood reflux overload against the influx of blood back flowing from aorta. This excessive wall stress results in myocardial fibrosis and ultimately systolic dysfunction [9]. Detecting myocardial fibrosis before a decline in LVEF is critical, as early intervention can prevent irreversible damage and improve both prognosis and quality of life. Once myocardial fibrosis progresses to the point of LVEF decline, surgical intervention is less effective in altering the clinical outcome. Therefore, identifying reliable markers to guide early surgical in asymptomatic patients is essential for risk stratification and reducing mortality [9,10,11].

Global longitudinal strain (GLS) measured via speckle tracking echocardiography evaluates the longitudinal shortening of the LV from base to apex. GLS is more sensitive than LVEF in detecting subtle impairments in systolic function, even when LVEF is preserved [11,12,13,14]. Due to its ability to identify subclinical myocardial dysfunction, GLS is being explored as a potential prognostic biomarker in asymptomatic aortic valvular heart disease with preserved EF [15,16,17]. However, the role of GLS specifically in chronic asymptomatic moderate to severe AR remains under investigation and requires stronger supporting evidence.

To date, only two systematic reviews have explored the prognostic value of GLS in chronic asymptomatic moderate to severe AR [18,19]. The first systematic review by deCampos et al. published in 2020 [18] included six AR-specific articles. The second, by Liao et al. in 2024 [19], included only three AR-related studies and focused more broadly on aortic valve disease, including aortic stenosis. Since then, additional studies have been published, highlighting the need for an updated systematic review. The aim of this systematic review and meta-analysis is to evaluate the prognostic utility of GLS in patients with chronic asymptomatic moderate to severe AR. Specifically, the review seeks to determine whether GLS can serve as an early marker of subclinical myocardial dysfunction, identify thresholds associated with adverse outcomes, and assess its potential to inform clinical decision-making regarding the timing of surgical intervention in this patient population. GLS) is derived from myocardial deformation imaging, most commonly using speckle-tracking echocardiography or, in some studies, cardiac magnetic resonance (CMR) feature-tracking. The GLS quantifies the percentage of longitudinal shortening from base to apex during systole, with more negative values indicating better systolic function. Typical acquisition standards include high-quality apical views and adequate frame rates to ensure reproducibility. As methodologies differ across vendors and platforms, inter-vendor variability and differences in post-processing algorithms may influence reported GLS values and cutoffs [20]. 

## 2. Materials and Methods

### 2.1. Protocol Registration

Ethical approval from an institutional review board was not required for this systematic review and meta-analysis, as the study involved no direct patient contact and utilized only publicly accessible data sources. The review process adhered to the Preferred Reporting Items for Systematic Reviews and Meta-Analyses (PRISMA) guidelines [21]. The completed PRISMA checklist can be found as the supplementary material. The study protocol was registered with PROSPERO (registration number CRD42024579540), an international database designed to promote transparency, prevent duplication of systematic reviews, and minimize reporting bias by allowing comparisons between the registered protocol and the final published review.

### 2.2. Databases and Study Search

Information was collected by two separate investigators and then finalized by the primary author. The search strategy was created by a librarian specialized in medical research utilizing the Peer-Reviewed Electronic Search Strategy (PRESS) guidelines [22]. The search strategy was initially developed for PubMed and employed search criteria specific to that database. Afterwards, it was then modified to suit subsequent searches on other databases, however overall modification needed was minimal. Searches were conducted on PubMed, 28 November 2024, Embase 28 November 2024, and Cochrane, 28 November 2024. Six studies from the previous systematic reviews [18,19] were additionally added in the search. The complete search strategy is available in Appendix A.

### 2.3. Eligibility Criteria

**Figure 1:**
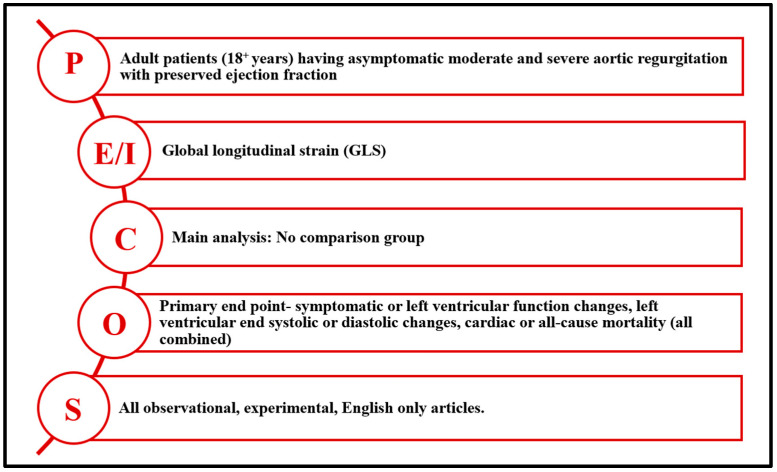
The PECOS (P—population; E/I—exposure/intervention; C—comparator; O—outcome; S—study design) framework for the eligibility criteria.

This systematic review followed the PECOS [23] framework (Population, Exposure, Comparator, Outcomes, and Study Design) as outlined in Figure 1. Studies were included if they were published in English prior to 28 November 2024, and met the criteria of being observational cohort studies, case series, randomized controlled trials (RCTs), or single-arm studies. For inclusion in the meta-analysis, studies were also required to report multivariate hazard ratios (HRs). When interpreting included studies, it is important to note that GLS acquisition and post-processing standards may vary across imaging platforms and vendors, which can introduce variability in measurement [20].

The study population consisted of adults over 18 years of age diagnosed with chronic asymptomatic moderate or severe aortic regurgitation and preserved LVEF. Studies involving populations that did not meet these criteria were excluded. Additionally, eligible studies needed to evaluate global longitudinal strain (GLS) as a marker of disease progression. This included outcomes such as the onset of symptoms, decline in LVEF, LVESD, LVEDD, number of cardiac mortalities, or all-cause mortality. Studies that did not assess GLS as an outcome or variable of interest were excluded. The review excluded non-English publications, single-patient case reports, conference abstracts or posters, animal studies, commentaries, opinion pieces, position papers, and editorials. Previously published meta-analyses and systematic reviews were also excluded to avoid redundancy.

### 2.4. Selection Process

All articles were directly imported into Rayyan to allow for organized article selection. Removal of duplicate articles was performed by the primary researcher (MK). All articles were reviewed by three independent reviewers (MK, TS, SW) through Rayyan who were blinded to each other’s decisions throughout each individual round of screening. The first round consisted of title screening only, followed by unblinding of the article selection. The three reviewers then met to discuss whichever articles had discrepancies in selection. Once a consensus on all articles with conflicting decisions was reached the selection process would move forward to the next round, in this case abstract screening. A similar process was followed leading to the final round of selection, full-text screening. After thorough analysis of each article and final discussion between the three reviewers, the remaining articles were included in the systematic review or meta-analysis.

### 2.5. Data Extraction

Remaining full-text articles were collected for data using a custom-designed data extraction form by three independent reviewers. Any disagreements regarding the collected data were discussed until absolute consensus was reached. Data consisted of study elements such as study title, the author with year, study design, population sample size, characteristics of study sample, and outcomes of interest. The characteristics of the study sample included age, male, bicuspid valve, left ventricular end-systolic diameter, and left ventricular end-diastolic diameter, the onset of symptoms, a decline in LVEF, LVESD, and LVEDD, number of cardiac mortalities, or all-cause mortality. The final included data was verified for accuracy by all investigators involved.

### 2.6. Assessment of Bias Risk

Risk assessment was performed using the National Heart, Lung and Blood Institute (NHLBI) quality assessment tool [24]. The types of the study design from each article were determined by the primary investigator and confirmed by a biostatistician. Two independent researchers (TS, DN) separately utilized the tool rating articles based on the guidelines provided by the NHLBI. Disagreements were discussed between both reviewers with final consensus determined by the primary investigator. Each article received a final rating based on the total of each individual component as delineated in the NHLBI quality assessment tool. Each article received a final rating based on the total of each individual component as delineated in the quality assessment tool’s guidelines. The number of “yes” answers utilized for the final rating of good (7–9), medium (4–6), or poor (≤3) given to each study. We assessed interrater agreement using percent agreement, as one reviewer did not use all available rating categories, rendering chance-corrected measures like Cohen’s Kappa or Gwet’s AC1 inappropriate due to lack of variability in the ratings [25].

### 2.7. Statistical Analysis

This meta-analysis was conducted to synthesize prognostic evidence from multiple studies evaluating cardiac-related risk factors across a range of clinical outcomes. Data were extracted from a structured summary table, which included hazard ratios (HRs), confidence intervals (CIs), event types, and relevant study characteristics. Both univariate and multivariate effect estimates were reviewed. This meta-analysis utilized the hazard ratio as the effect size index. For the meta-analysis, we included only those studies that reported multivariate hazard ratios along with their 95% confidence intervals, as these were consistently available across the majority of studies. In addition to confidence intervals, prediction intervals were calculated to quantify the expected range of true effects in future comparable studies. While confidence intervals reflect the precision of the pooled estimate, prediction intervals offer a more realistic estimate of the dispersion of effect sizes across different settings, populations, or study designs highlighting the uncertainty and potential variability in real-world applications [26]. For each study, the natural logarithm of the hazard ratio (log-HR) was calculated, and the corresponding standard error (SE) was derived from the reported confidence interval using standard meta-analytic methods [27,28,29,30,31]. 

These log-HRs and their variances were then pooled using a random-effects model based on the DerSimonian–Laird method to account for between-study heterogeneity [27]. Heterogeneity was assessed using the I^2^ statistic, which represents the percentage of total variation across studies due to heterogeneity rather than chance, and Tau^2^, which estimates the between-study variance [28]. A forest plot was generated to visualize the individual and pooled estimates, with HR = 1 used as the null reference line. To assess potential publication bias, we conducted Egger’s regression test, which evaluates asymmetry in a funnel plot of study effect sizes against their precision. Specifically, the standard normal deviate (SND) of each study’s log-transformed hazard ratio was regressed on the inverse of its standard error [29,30,31]. Sensitivity and subgroup analyses were not conducted due to the limited number of included studies (n = 4), which would render such analyses statistically underpowered and potentially unreliable [32].

## 3. Results

### 3.1. Study Screening

**Figure 2:**
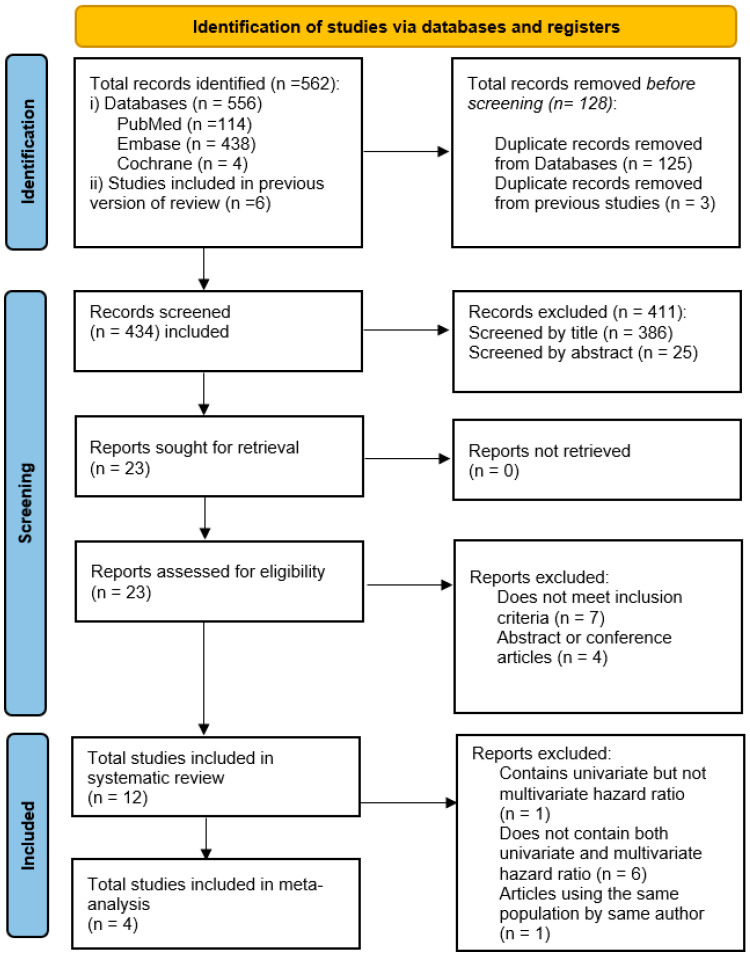
PRISMA (Preferred Reporting Items for Systematic Reviews and Meta-Analysis) flow diagram detailing the disposition of screened, included, and excluded studies.

Three databases (PubMed, Embase, and Cochrane) were used for our systematic search. Total of 556 articles were identified from three databases (114 in PubMed, 438 in Embase, and 4 in Cochrane), and 6 studies included in the previous version of review were added to the initial records, yielding 562 articles in total. One hundred and twenty-five duplicate studies were removed from the three databases, and 3 from the previous version of review, yielding 128 total removed duplicate records, leaving 434 articles for the initial screening. Afterwards, 386 articles were excluded after title screening, and later 25 articles were excluded after abstract screening, which left 23 articles. All 23 articles were retrieved for the final full-text screening. Out of 23 articles, 7 articles were excluded since these did not meet inclusion criteria, and 4 articles were excluded since these articles were either abstract or conference articles. In the end, 12 articles were included for the final systematic review. Out of the articles selected for the systematic review, one article was excluded for meta-analysis since it contained univariate hazard ratio but not multivariate hazard ratio. 6 articles were excluded since these articles did not contain both univariate and multivariate hazard ratio. Of note, two articles were published under the same author, Alashi, and these two articles were conducted under the same sample population. Therefore, only the latest article published in 2020 was considered for the final meta-analysis. Four articles were selected for the final meta-analysis. The screening process is shown in Figure 2.

### 3.2. Study Quality

The final studies were evaluated for the quality scores with the NHLBI quality assessment tool, which are provided in Table 1. Two reviewers (TS, SW) participated in the quality assessment. The inter-rater agreement was 83.3%. None of the studies were of poor quality (score range ≤ 3).

The systematic review includes 12 studies, most of which are observational cohort studies (OCS), along with a few case series (CSS) and one case–control study (CCS). Sample sizes varied widely, ranging from 80 to 1063 participants, with study populations generally composed of middle-aged to older adults (mean ages between 45 and 70 years). Across studies, the majority of participants were male, often comprising over 60% of the sample. Bicuspid aortic valve was reported in about half of the studies, with prevalence ranging from 20% to 71%. Clinical characteristics commonly included left ventricular end-systolic and end-diastolic diameters (LVESD and LVEDD), although indexed values (LVESDi, LVEDDi) were frequently not reported. Key outcomes tracked across studies included the development of symptoms, reduced LVEF, need for aortic valve intervention, and cardiovascular or all-cause mortality. However, not all studies reported on every outcome, particularly mortality-related measures (Table 1). Across studies reporting diagnostic thresholds, GLS cutoffs ranged from approximately −16% to −18.5%, with moderate sensitivity and specificity. Table 2 details, for each study, the imaging modality, measurement convention, baseline population severity, and predicted outcome. Notably, thresholds derived from different modalities (CMR feature-tracking vs. Two-dimensional speckle-tracking echocardiography) and cohorts limit direct comparability. Notably, some studies reported GLS as categorical thresholds (e.g., −17% to −18.5%), while others analyzed GLS as a continuous variable per 1% absolute worsening. This variability highlights the lack of a standardized reporting framework, which may limit comparability of pooled estimates.

### 3.3. Meta-Analysis

**Figure 3:**
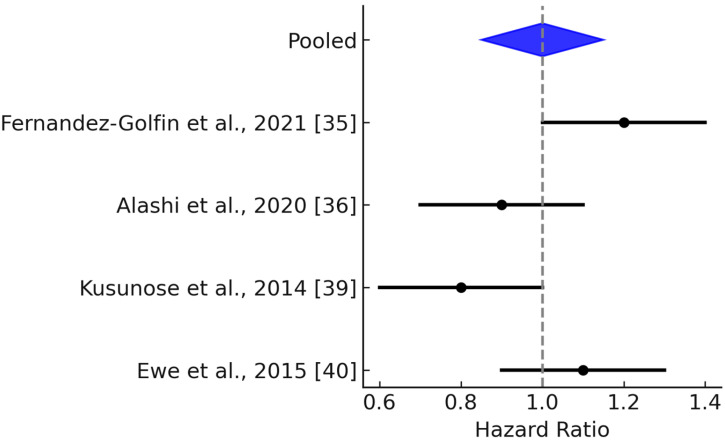
Forest plot showing pooled hazard ratios.

**Figure 4:**
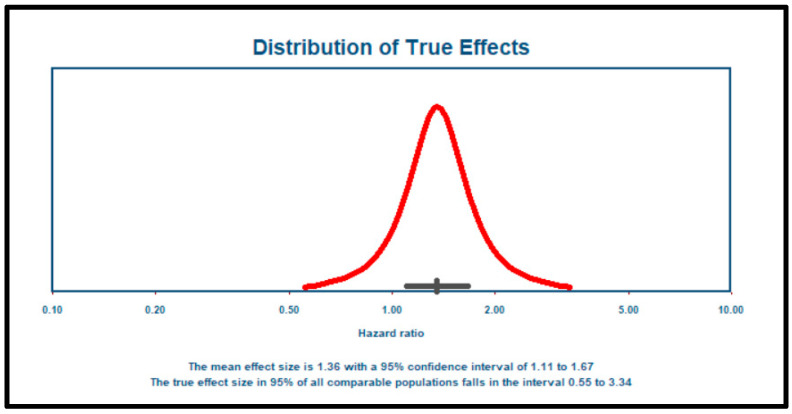
Distribution of true effects.

The follow-up durations across the four studies included in the meta-analysis ranged from approximately 3 to 7 years, providing mid- to long-term prognostic context. Specifically, Alashi et al. (2020) reported a median follow-up of 6.6 years (IQR: 5.2–9.1 years) [36]; Verseckaite et al. (2018) reported a 5-year follow-up of 4.7 ± 2.6 years [37]; and Ewe et al. (2015) reported a mean follow-up of 4.2 ± 3.2 years [40]. To authors’ best knowledge, Reil et al. (2020) did not report the follow-up duration [41]. In this meta-analysis of 1125 patients from four studies [36,37,40,41], the mean effect size was 1.359, with a 95% confidence interval of 1.106 to 1.668, indicating a statistically significant association (Figure 3). The Z-test result (Z = 2.926, *p* = 0.003) further confirmed that the mean effect size is significantly different from zero. Heterogeneity analysis revealed a Q-statistic of 13.244 (*p* = 0.004) and an I^2^ value of 77%, suggesting considerable variability in effect sizes likely due to real differences across studies. The tau-squared (τ^2^) value was 0.033, and tau (τ) was 0.181, indicating moderate between-study variability. The 95% prediction interval, ranging from 0.553 to 3.340, highlights the potential variation in effect sizes in future comparable studies (Figure 4). However, since only four studies were included, the findings particularly estimate of heterogeneity should be interpreted with caution.

### 3.4. Publication Bias or Small-Study Effects

**Figure 5:**
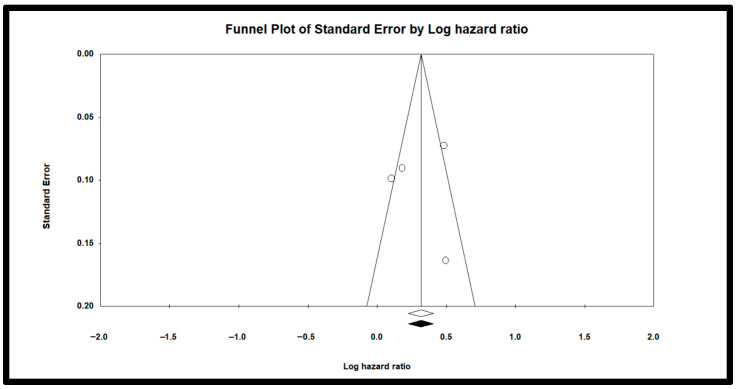
Funnel plot to visually inspect publication bias. Dots represent individual study estimates; the vertical line indicates the pooled effect, and the diagonal lines show expected 95% confidence limits around the pooled estimate.

A regression analysis (Egger’s test) yielded an intercept of –0.97 (*p* = 0.47), suggesting no statistically significant evidence of publication bias. However, given the small number of included studies (n = 4), this test may be underpowered to detect true asymmetry. Visually, the funnel plot (Figure 5) shows some degree of asymmetry, which could reflect publication bias or small-study effects, though funnel plots are also considered unreliable when fewer than 10 studies are included. Taken together, these findings highlight the need for cautious interpretation.

## 4. Discussion

This systematic review and meta-analysis aimed to evaluate the prognostic utility of global longitudinal strain (GLS) in patients with chronic asymptomatic moderate to severe aortic regurgitation (AR) with preserved ejection fraction. The primary goal was to assess whether GLS could serve as an early marker of subclinical myocardial dysfunction and predict key outcomes such as symptom onset, decline in LVEF, left ventricular dilation, and mortality. Our findings support the growing body of evidence that GLS is associated with adverse outcomes in asymptomatic patients with preserved EF.

Some studies, such as Reil et al. and Ewe et al., obtained GLS values at the time when patients already developed the indications for AV surgery [40,41]. Reil et al. [41] reported that compared to severe AR patients without indications for AV surgery, severe AR patients with symptoms or reduced LVEF had worse GLS. Ewe et al. [40] reported that although LVEF remained preserved, symptomatic moderate to severe AR groups had lower GLS than asymptomatic groups. These two studies confirm that once patients develop the indications of AV surgery, GLS is already impaired even if LVEF remains normal. However, this does not suggest that GLS can predict the development of the indications for AV surgery since GLS was obtained when patients already developed the indications for AV surgery in these studies, not prior.

On the other hand, compared to the above studies, other studies such as Kočková et al., Martín et al., Fernández-Golfín et al., Alashi et al., Verseckaite et al., Zeng et al., Kusunose et al., the subgroup analysis of Ewe et al., and Li et al., obtained the baseline GLS at the time when patients were asymptomatic with preserved EF and used this baseline GLS as a reference for prognostic value [33,34,35,36,37,38,39,40,42]. Overall, these studies demonstrate that GLS can predict the prognosis. Kočková et al., Verseckaite et al., Zeng et al., Kusunose et al., and the subgroup analysis of Ewe et al. demonstrated that impaired baseline GLS was predictive of the eventual development of symptoms or reduced LVEF, aligning with the rationale that GLS detects subclinical dysfunction before irreversible damage occurs [34,37,39,40,44]. These findings suggest that the baseline GLS obtained in asymptomatic and preserved LVEF period can predict the future manifestation of new symptoms and reduced LVEF. Notably, Kočková et al. also found GLS to be superior to LVESD in predicting deterioration in LVEF, highlighting the potential of GLS to complement or even replace current structural criteria for surgical referral [33].

While our meta-analysis showed a significant pooled hazard ratio indicating prognostic relevance of impaired GLS, heterogeneity across studies was high (I^2^ = 77%), and the prediction interval was wide. This suggests that the true effect of GLS may vary significantly across clinical settings, underscoring the importance of individualized interpretation and the need for standardized GLS thresholds. In contrast, a few studies yielded contradictory results. For instance, Suzuki et al. did not find a clear association between GLS and postoperative prognosis [43], while Martín et al. and Fernández-Golfín et al. reported that GLS was not statistically significant in multivariate models, despite showing separation in Kaplan–Meier curves in Fernández-Golfín et al. [33,35]. These discrepancies may be explained by methodological differences, including varying definitions of abnormal GLS, differences in timing of GLS measurement, and heterogeneity in endpoints. Furthermore, some studies included patients already undergoing surgery, blurring the temporal relationship between GLS and disease progression.

In terms of structural progression, our review also observed a consistent relationship between impaired GLS and LV dilation metrics. Studies by Reil et al. and Li et al. indicated that worsening GLS was associated with increased ventricular dimensions [41,42], suggesting that strain imaging may serve as a more sensitive and continuous marker of remodeling, compared to traditional static measurements. Of note, Li et al. did not investigate either LVESD or LVEDD, but instead this study studied LVEDV in relation to GLS in AR groups including all levels of severity.

Mortality outcomes, while less frequently reported, added further context. Studies by Alashi et al. [36,38] found that both pre- and postoperative GLS values were associated with long-term survival, reinforcing the prognostic relevance of strain imaging beyond structural deterioration. However, the overlap of study populations between these publications limits the strength of this evidence. Still, the trend supports further investigation into GLS as a long-term prognostic tool, not only for timing intervention but also for postoperative monitoring. In terms of aortic stenosis and mitral regurgitation, several systematic review and meta-analysis showed that preoperative GLS was associated with post-operative clinical outcomes including death and change in LVEF [45,46,47,48]. The fact that GLS can detect the reverse remodeling of left ventricular function and its effect on outcomes such as mortality and morbidity in other valvular disease promises the role of GLS in the post-op prognosis in AR, but further study is needed.

Alashi et al. also stated that the long-term mortality was not significantly higher in the subgroup with better LV-GLS and who did not undergo the AV surgery compared to better LV-GLS and who underwent the AV surgery [38]. This implies that the AV surgery can be deferred until a certain GLS threshold. Therefore, identifying the cutoff value of GLS for surgical referral is another important topic for future study. From our systematic review, Fernández-Golfín et al. stated that the ideal cutoff is −16% with sensitivity of 68.8 with specificity of 70.6 [35]. Verseckaite et al. stated that GLS worse than −18.5% was reliable in detecting the deterioration of LVEF with sensitivity of 83% and specificity of 84% [37], and Ewe et al. reported that GLS value of −17.4% showed the highest sensitivity of 77% and specificity of 57% [40].

Transportability of GLS thresholds to asymptomatic moderate to severe AR is limited by modality- and cohort-specific factors. The thresholds reported here (≈−16% to −18.5%) were derived using different imaging platforms (CMR feature-tracking vs. Two-dimensional STE), vendor algorithms, and baseline populations (e.g., ‘significant’ AR vs. strictly asymptomatic moderate–severe AR), and were modeled as signed values (more negative indicates better systolic function). Given known inter-vendor variability and acquisition differences, applying any single cutoff universally, particularly to asymptomatic severe AR risks misclassification. Standardized acquisition, vendor-independent post-processing, and prospective, disease-stage–specific validation is needed before thresholds can be generalized across settings.

As well as the prognosis of moderate to severe AR, several articles studied the role of GLS in the progression of the severity of AR. Our systematic review showed that the worsening GLS overall has correlation with the deterioration of the severity of AR. Reil et al. reported that compared to control groups, GLS was worse in severe AR regardless of with and without indication for surgery despite normal LVEF [42]. Li et al. also reported that AR group has worse GLS value than control group [42]. Likewise, Fernández-Golfín reported that compared to the control group, AR group has worse GLS despite all subjects remaining normal LVEF [35]. Verseckaite et al. was the only study that showed ambiguous results [37]. This study showed that severe AR had significantly lower GLS than control, but moderate AR was not. While our meta-analysis suggests that impaired GLS has prognostic value in asymptomatic AR, the small number of eligible studies and substantial heterogeneity mean that these findings should be interpreted as exploratory. Therefore, the role of GLS should currently be viewed as hypothesis-generating, pending further validation in larger prospective cohorts.

## 5. Strengths and Limitations

A key strength of this study lies in its focus on a clinically underexplored but important subgroup patient with asymptomatic moderate to severe AR and preserved EF. By synthesizing data across multiple studies and applying rigorous meta-analytic methods, we were able to assess the prognostic role of GLS within this niche yet high-risk population. The inclusion of prediction intervals further adds to the generalizability of findings by offering insight into the potential variability of GLS effects in future clinical settings. However, this study is not without limitations. One of the most significant limitations is that hazard ratios were often reported for combined composite outcomes, rather than stratified by specific clinical endpoints such as symptom onset, reduced LVEF, or mortality. This aggregation limits the ability to interpret which outcomes GLS is most predictive of. Additionally, the small number of studies eligible for meta-analysis (n = 4) reduced the statistical power and increased susceptibility to bias. The heterogeneity in study designs, outcome definitions, and GLS cutoff values also limits the comparability and generalizability of findings. Most included studies were observational, making them prone to confounding, and overlapping patient datasets in some publications may have artificially inflated associations. Given that only four studies were eligible for pooling, the combined effect size should be interpreted cautiously and considered exploratory rather than definitive.

In addition to the methodological heterogeneity across studies, several technical limitations inherent to speckle-tracking echocardiography must be considered. First, inter-vendor variability in acquisition and post-processing algorithms may lead to inconsistent GLS measurements across different ultrasound systems. Second, GLS is dependent on frame rate settings, and suboptimal frame rates can reduce accuracy and reproducibility. Finally, basal longitudinal strain measurements may be strongly influenced by chest wall morphology, particularly in individuals with anterior sternal depression or pectus excavatum, which can distort imaging windows and compromise measurement reliability [49,50,51]. Additionally, variability in threshold derivation (modality, vendor, and cohort) suggests that currently reported GLS cutoffs are context-dependent and not directly transportable to all asymptomatic severe AR populations. These limitations underscore the need for standardized acquisition protocols and vendor-independent software to ensure the robustness and generalizability of GLS as a prognostic biomarker.

Another limitation is that although meta-regression could help explore sources of heterogeneity, we did not perform this analysis because only four studies were included in the meta-analysis. Pooling results from only four studies with substantial heterogeneity may limit the reliability of the combined effect size; thus, our findings should be regarded as hypothesis-generating. Conducting meta-regression with such a small number of studies produces unstable and unreliable estimates, as highlighted in the Cochrane Handbook for Systematic Reviews of Interventions [52]. This restriction limits our ability to formally test which study-level factors (e.g., GLS thresholds, imaging modality, timing of measurement) may account for the observed heterogeneity. Future reviews with larger datasets will be able to explore sources of heterogeneity more robustly using meta-regression. Another limitation is variability in how GLS was modeled across studies (continuous per 1% worsening vs. categorical thresholds). This heterogeneity in measurement scales may affect comparability of pooled HRs, and our inability to harmonize across definitions reflects the urgent need for standardized GLS reporting in future studies.

## 6. Conclusions

GLS appears to be a promising biomarker for detecting subclinical dysfunction in patients with chronic asymptomatic moderate to severe AR and preserved LVEF. Our findings suggest that impaired GLS is associated with adverse outcomes and may enhance early risk stratification. However, because only four studies were eligible for meta-analysis and heterogeneity was substantial (I^2^ = 77%), these results should be interpreted with caution and considered hypothesis-generating rather than definitive. Importantly, while GLS may complement existing markers of disease progression, the current evidence does not establish its role in determining the optimal timing for surgical intervention. GLS is promising for early risk stratification in asymptomatic AR; however, clinically actionable thresholds remain context-dependent, and prospective multicenter studies with standardized methods are required before a universal cutoff can be recommended.

## Figures and Tables

**Table 1:** Characteristics of studies included in the systematic review (N = 12).

Author, Year	Study Design	Sample Size	Characteristics of the Entire Sample					Outcomes (Number)					Quality Rating
			Age *M* (*SD*) or [*Range*]	Male *n* (%)	Bicuspid Valve *n* (%)	LVESD (mm)/ LVESDi (cm/m^2^) *M* (*SD*)	LVEDD (mm)/ LVEDDi (cm/m^2^) *M* (*SD*)	Dev. of SX (n)	Reduced LVEF (n)	Aortic Valve Interv. (n)	CV Mortality (n)	All-Cause Mortality (n)	
Garcia Martin et al., 2022 [33]	OCS	126	70.1 (17.2)	75 (59.5%)	25 (20.5%)	32.5 (6.4)/ NR	52.5 (7.4)/ NR	5	NR	25	4	NR	Medium
Kočková et al., 2022 [34]	OCS	127	45 (14)	107 (84%)	90 (71%)	37 (5)/ NR	58 (6)/ NR	34	7	41	NR	NR	Good
Fernandez-Golfin et al., 2021 [35]	OCS	109	* 57.42 (15.8)	74 (68%)	NR	NR/ NR	NR/ NR	9	4	14	0	1	Good
Alashi et al., 2020 [36]	OCS	865	52 (15)	684 (79%)	NR	NR/ 1.9 (0.4)	NR/ 2.8 (0.5)	NR	NR	NR	94	105	Good
Verseckaite et al., 2018 [37]	OCS	127	* 46.6 (15.2)	88 (69%)	NR	* 34.84 (5.5)/NR	* 51.68 (5.8) /NR	4	12	NR	NR	NR	Good
Alashi et al., 2017 [38]	OCS	1063	53 (16)	813 (77%)	383 (36%)	35 (6)/ 1.7 (0.3)	54 (8)/ 2.7 (0.5)	NR	NR	NR	135	146	Good
Kusunose et al., 2014 [39]	OCS	159	50 (15)	124 (80%)	70 (45%)	37 (6)/ NR	57 (7)/ NR	41	28	50	0	0	Good
Ewe et al., 2015 [40]	CSS	129	* 54.5 (16.5)	82 (64%)	40 (31%)	* 35 (7)/ NR	* 56 (8)/ NR	21	5	26	NR	NR	Good
Reil et al., 2020 [41]	CCS	80	* 57.5 (15.1)	66 (83%)	26 (43%)	NR/ * 2.02 (0.4)	NR/ NR	NR	NR	NR	NR	NR	Good
Li et al., 2013 [42]	CSS	107	* 52.3 (15.5)	59 (55%)	NR	NR/ NR	NR/ NR	NR	NR	NR	NR	NR	Good
Suzuki et al., 2024 [43]	OCS	210	65 [46–73]	148 (71%)	NR	41 (6)/ NR	61 (7)/ NR	4	NR	33	0	3	Good
Zeng et al., 2021 [44]	OCS	176	* 56.5 (12.8)	70 (40%)	NR	NR/ NR	NR/ NR	NR	NR	NR	NR	NR	Good

* Indicates reviewer-calculated values based on combining subgroup means reported in the original article; significant AR (defined as moderate to severe AR); LVESD = Non-indexed Left Ventricular End Systolic Diameter; LVESDi = Indexed Left Ventricular End Systolic Diameter; LVEDD = Non-indexed Left Ventricular End Diastolic Diameter; LVEDDi = Indexed Left Ventricular End Diastolic Diameter; SX = Symptoms; LVEF = Left Ventricular Ejection Fraction; CV = Cardiovascular; OCS = Observational Cohort Study; CSS = Case Series Study; CCS = Case–Control Study; NR = Not Reported.

**Table 2:** Reported GLS cutoff values, diagnostic accuracy, and modeling approach for predicting adverse outcomes in asymptomatic moderate to severe aortic regurgitation.

Study (Year)	GLS Cutoff Value	Sensitivity (%)	Specificity (%)	Outcome Predicted	Modeling Approach
Alashi et al., 2020 [36]	NA	NA	NA	Adverse outcomes (composite)	Continuous (per 1% worsening, absolute value)
Verseckaite et al., 2018 [37]	–18.5%	83	84	Reduced LVEF	Categorical (cutoff)
Ewe et al., 2015 [40]	–17.4%	77	57	Symptom onset/adverse outcome	Categorical (cutoff)
Reil et al., 2020 [41]	NA	NA	NA	Surgical indication/subgroup outcomes	Continuous (absolute values)

NA = not applicable; GLS = global longitudinal strain; LVEF = left ventricular ejection fraction. The variability in GLS cutoff thresholds and associated diagnostic accuracy underscores the lack of a standardized benchmark across studies. This heterogeneity highlights the need for prospective multicenter trials to establish clinically meaningful GLS thresholds in asymptomatic aortic regurgitation.

## Supplementary Materials

The following supporting information can be downloaded at: https://www.mdpi.com/article/10.3390/jcm14186534/s1, Reporting checklist for systematic review.

## Appendix A. Search Strategies (Search Date: 28 November 2024)

**Table A1:** Search Strategies Used in this Review.

Database	Query Used	Limit Criteria	Results	Totals
PubMed				
#1	Aortic regurgitation	Inception—11/28/2024	31,031	
#2	Mixed aortic valve disease	Inception—11/28/2024	782	
#3	#1 OR #2	Inception—11/28/2024	31,394	
#4	Global Longitudinal Strain	Inception—11/28/2024	7342	
#5	GLS	Inception—11/28/2024	5858	
#6	#4 OR #5	Inception—11/28/2024	9861	
#7	#3 AND #6	Inception—11/28/2024	114	
Embase				
#1	Aortic regurgitation	Inception—11/28/2024	57,763	
#2	Mixed aortic valve disease	Inception—11/28/2024	1272	
#3	#1 OR #2	Inception—11/28/2024	58,275	
#4	Global Longitudinal Strain	Inception—11/28/2024	14,412	
#5	GLS	Inception—11/28/2024	12,609	
#6	#4 OR #5	Inception—11/28/2024	18,851	
#7	#3 AND #6	Inception—11/28/2024	438	
Cochrane Library				
#1	Aortic regurgitation	Title Abstract Keyword; Inception—11/28/2024	716	
#2	Mixed aortic valve disease	Title Abstract Keyword; Inception—11/28/2024	61	
#3	#1 OR #2	Title Abstract Keyword; Inception—11/28/2024	762	
#4	Global Longitudinal Strain	Title Abstract Keyword; Inception—11/28/2024	828	
#5	GLS	Title Abstract Keyword; Inception—11/28/2024	618	
#6	#4 OR #5	Title Abstract Keyword; Inception—11/28/2024	1052	
#7	#3 AND #6	Title Abstract Keyword; Inception—11/28/2024	4	
Previous Systematic Review			6	
			GRAND TOTAL	562

## References

[1] Gössl M., Stanberry L., Benson G., Steele E., Garberich R., Witt D., Cavalcante J., Sharkey S., Enriquez-Sarano M. (2023). Burden of Undiagnosed Valvular Heart Disease in the Elderly in the Community: Heart of New Ulm Valve Study. JACC Cardiovasc. Imaging.

[2] Otto C.M., Nishimura R.A., Bonow R.O., Carabello B.A., Erwin J.P., Gentile F., Jneid H., Krieger E.V., Mack M., McLeod C. (2021). 2020 ACC/AHA guideline for the management of patients with valvular heart disease: A report of the American College of Cardiology/American Heart Association Joint Committee on Clinical Practice Guidelines. Circulation.

[3] Aronow W.S., Ahn C., Kronzon I., Nanna M. (1994). Prognosis of patients with heart failure and unoperated severe aortic valvular regurgitation and relation to ejection fraction. Am. J. Cardiol..

[4] Dujardin K.S., Enriquez-Sarano M., Schaff H.V., Bailey K.R., Seward J.B., Tajik A.J. (1999). Mortality and morbidity of aortic regurgitation in clinical practice: A long-term follow-up studies. Circulation.

[5] Chaliki H.P., Mohty D., Avierinos J.-F., Scott C.G., Schaff H.V., Tajik A.J., Enriquez-Sarano M. (2002). Outcomes after aortic valve replacement in patients with severe aortic regurgitation and markedly reduced left ventricular function. Circulation.

[6] Wang Y., Shi J., Li F., Wang Y., Dong N. (2016). Aortic valve replacement for severe aortic regurgitation in asymptomatic patients with normal ejection fraction and severe left ventricular dilatation. Interact. Cardiovasc. Thorac. Surg..

[7] Yang L.-T., Enriquez-Sarano M., Michelena H.I., Nkomo V.T., Scott C.G., Bailey K.R., Oguz D., Ullah M.W., Pellikka P.A. (2019). Predictors of Progression in Patients with Stage B Aortic Regurgitation. J. Am. Coll. Cardiol..

[8] Siani A., Perone F., Costantini P., Rodolfi S., Muscogiuri G., Sironi S., Carriero S., Pavon A.G., van der Bilt I., van Rosendael P. (2022). Aortic regurgitation: A multimodality approach. J. Clin. Ultrasound.

[9] Akinseye O.A., Pathak A., Ibebuogu U.N. (2018). Aortic Valve Regurgitation: A Comprehensive Review. Curr. Probl. Cardiol..

[10] Lee J.K., Franzone A., Lanz J., Siontis G.C., Stortecky S., Gräni C., Roost E., Windecker S., Pilgrim T. (2018). Early Detection of Subclinical Myocardial Damage in Chronic Aortic Regurgitation and Strategies for Timely Treatment of Asymptomatic Patients. Circulation.

[11] Smedsrud M.K., Pettersen E., Gjesdal O., Svennevig J.L., Andersen K., Ihlen H., Edvardsen T. (2011). Detection of left ventricular dysfunction by global longitudinal systolic strain in patients with chronic aortic regurgitation. J. Am. Soc. Echocardiogr..

[12] Abou R., van der Bijl P., Bax J.J., Delgado V. (2020). Global longitudinal strain: Clinical use and prognostic implications in contemporary practice. Heart.

[13] Klaeboe L.G., Edvardsen T. (2019). Echocardiographic assessment of left ventricular systolic function. J. Echocardiogr..

[14] Stokke T.M., Hasselberg N.E., Smedsrud M.K., Sarvari S.I., Haugaa K.H., Smiseth O.A., Edvardsen T., Remme E.W. (2017). Geometry as a Confounder When Assessing Ventricular Systolic Function: Comparison Between Ejection Fraction and Strain. J. Am. Coll. Cardiol..

[15] Azevedo C.F., Nigri M., Higuchi M.L., Pomerantzeff P.M., Spina G.S., Sampaio R.O., Tarasoutchi F., Grinberg M., Rochitte C.E. (2010). Prognostic significance of myocardial fibrosis quantification by histopathology and magnetic resonance imaging in patients with severe aortic valve disease. J. Am. Coll. Cardiol..

[16] Li C.-M., Li C., Bai W.-J., Zhang X.-L., Tang H., Qing Z., Li R. (2013). Value of three-dimensional speckle-tracking in detecting left ventricular dysfunction in patients with aortic valvular diseases. J. Am. Soc. Echocardiogr..

[17] Purwowiyoto S.L., Halomoan R. (2022). Highlighting the role of global longitudinal strain assessment in valvular heart disease. Egypt. Heart J..

[18] deCampos D., Teixeira R., Saleiro C., Botelho A., Gonçalve L. (2020). Global longitudinal strain in chronic asymptomatic aortic regurgitation: Systematic review. Echo Res. Pract..

[19] Liao H., Yang S., Yu S., Hu X., Meng X., Wu K. (2024). Prognostic Value of Left Ventricular Global Longitudinal Strain for Major Adverse Cardiovascular Events in Patients with Aortic Valve Disease: A Meta-Analysis. Cardiology.

[20] Gherbesi E., Gianstefani S., Angeli F., Ryabenko K., Bergamaschi L., Armillotta M., Guerra E., Tuttolomondo D., Gaibazzi N., Squeri A. (2024). Myocardial strain of the left ventricle by speckle tracking echocardiography: From physics to clinical practice. Echocardiography.

[21] Page M.J., McKenzie J.E., Bossuyt P.M., Boutron I., Hoffmann T.C., Mulrow C.D., Shamseer L., Tetzlaff J.M., Akl E.A., Brennan S.E. (2021). The PRISMA 2020 statement: An updated guideline for reporting systematic reviews. BMJ.

[22] McGowan J., Sampson M., Salzwedel D.M., Cogo E., Foerster V., Lefebvre C. (2016). PRESS Peer Review of Electronic Search Strategies: 2015 Guideline Statement. J. Clin. Epidemiol..

[23] Morgan R.L., Whaley P., Thayer K.A., Schünemann H.J. (2018). Identifying the PECO: A framework for formulating good questions to explore the association of environmental and other exposures with health outcomes. Environ. Int..

[24] National Heart, Lung, and Blood Institute (2024). Study Quality Assessment Tools. https://internet-prod.nhlbi.nih.gov/health-topics/study-quality-assessment-tools.

[25] Li M., Gao Q., Yu T. (2023). Kappa statistic considerations in evaluating inter-rater reliability between two raters: Which, when and context matters. BMC Cancer.

[26] IntHout J., Ioannidis J.P.A., Rovers M.M., Goeman J.J. (2016). Plea for routinely presenting prediction intervals in meta-analysis. BMJ Open.

[27] DerSimonian R., Laird N. (2015). Meta-analysis in clinical trials revisited. Contemp. Clin. Trials.

[28] Higgins J.P., Thompson S.G. (2002). Quantifying heterogeneity in a meta-analysis. Stat. Med..

[29] Egger M., Davey Smith G., Schneider M., Minder C. (1997). Bias in meta-analysis detected by a simple, graphical test. BMJ.

[30] Sterne J.A., Egger M. (2001). Funnel plots for detecting bias in meta-analysis: Guidelines on choice of axis. J. Clin. Epidemiol..

[31] Sterne J.A.C., Sutton A.J., Ioannidis J.P.A., Terrin N., Jones D.R., Lau J., Carpenter J., Rücker G., Harbord R.M., Schmid C.H. (2011). Recommendations for examining and interpreting funnel plot asymmetry in meta-analyses of randomized controlled trials. BMJ.

[32] Deeks J.J., Higgins J.P.T., Altman D.G., Higgins J.P.T., Thomas J., Chandler J., Cumpston M., Li T., Page M.J., Welch V.A. (2019). Analysing data and undertaking meta-analyses. Cochrane Handbook for Systematic Reviews of Interventions.

[33] Martín A.G., Sequeiros M.A., Gómez A.G.G., Díaz L.M.R., Ruiz J.M.M., Baydés R.H., Mur J.L.M., Zamorano J.L., Fernández-Golfín C. (2022). Prognostic value of diastolic function parameters in significant aortic regurgitation: The role of the left atrial strain. J. Echocardiogr..

[34] Kočková R., Línková H., Hlubocká Z., Mědílek K., Tuna M., Vojáček J., Skalský I., Černý Š., Malý J., Hlubocký J. (2022). Multiparametric Strategy to Predict Early Disease Decompensation in Asymptomatic Severe Aortic Regurgitation. Circ. Cardiovasc. Imaging.

[35] Fernández-Golfín C., Hinojar-Baydes R., González-Gómez A., Monteagudo J.M., Esteban A., Alonso-Salinas G., Fernández M.A., García-Martín A., Santoro C., Pascual-Izco M. (2021). Prognostic implications of cardiac magnetic resonance feature tracking derived multidirectional strain in patients with chronic aortic regurgitation. Eur. Radiol..

[36] Alashi A., Khullar T., Mentias A., Gillinov A.M., Roselli E.E., Svensson L.G., Popovic Z.B., Griffin B.P., Desai M.Y. (2020). Long-Term Outcomes After Aortic Valve Surgery in Patients with Asymptomatic Chronic Aortic Regurgitation and Preserved LVEF: Impact of Baseline and Follow-Up Global Longitudinal Strain. JACC Cardiovasc. Imaging.

[37] Verseckaite R., Mizariene V., Montvilaite A., Auguste I., Bieseviciene M., Laukaitiene J., Jonkaitiene R., Jurkevicius R. (2018). The predictive value of left ventricular myocardium mechanics evaluation in asymptomatic patients with aortic regurgitation and preserved left ventricular ejection fraction. A long-term speckle-tracking echocardiographic study. Echocardiography.

[38] Alashi A., Mentias A., Abdallah A., Feng K., Gillinov A.M., Rodriguez L.L., Johnston D.R., Svensson L.G., Popovic Z.B., Griffin B.P. (2018). Incremental Prognostic Utility of Left Ventricular Global Longitudinal Strain in Asymptomatic Patients with Significant Chronic Aortic Regurgitation and Preserved Left Ventricular Ejection Fraction. JACC Cardiovasc. Imaging.

[39] Kusunose K., Agarwal S., Marwick T.H., Griffin B.P., Popović Z.B. (2014). Decision making in asymptomatic aortic regurgitation in the era of guidelines: Incremental values of resting and exercise cardiac dysfunction. Circ. Cardiovasc. Imaging.

[40] Ewe S.H., Haeck M.L., Ng A.C., Witkowski T.G., Auger D., Leong D.P., Abate E., Marsan N.A., Holman E.R., Schalij M.J. (2015). Detection of subtle left ventricular systolic dysfunction in patients with significant aortic regurgitation and preserved left ventricular ejection fraction: Speckle tracking echocardiographic analysis. Eur. Heart J. Cardiovasc. Imaging.

[41] Reil J.-C., Reil G.-H., Hecker N., Sequeira V., Borer J.S., Stierle U., Lavall D., Marquetand C., Busch C., Patzelt J. (2021). Reduced left ventricular contractility, increased diastolic operant stiffness and high energetic expenditure in patients with severe aortic regurgitation without indication for surgery. Interact. Cardiovasc. Thorac. Surg..

[42] Gao L., Lin Y., Ji M., Wu W., Li H., Qian M., Zhang L., Xie M., Li Y. (2022). Clinical Utility of Three-Dimensional Speckle-Tracking Echocardiography in Heart Failure. J. Clin. Med..

[43] Suzuki S., Amano M., Nakagawa S., Irie Y., Moriuchi K., Okada A., Kitai T., Amaki M., Kanzaki H., Nishimura K. (2024). Outcomes of Watchful Waiting Strategy and Predictors of Postoperative Prognosis in Asymptomatic or Equivocally Symptomatic Chronic Severe Aortic Regurgitation with Preserved Left Ventricular Function. J. Am. Heart Assoc..

[44] Zeng Q., Wang S., Zhang L., Li Y., Cheng L., Wang J., Yang Y., Wang D., Zhang Y., Xie Y. (2021). Left Ventricular Remodeling and Its Progression in Asymptomatic Patients with Chronic Aortic Regurgitation: Evaluation by Speckle-Tracking Echocardiography. J. Am. Soc. Echocardiogr..

[45] Stens N.A., van Iersel O., Rooijakkers M.J., van Wely M.H., Nijveldt R., Bakker E.A., Rodwell L., Pedersen A.L., Poulsen S.H., Kjønås D. (2023). Prognostic Value of Preprocedural LV Global Longitudinal Strain for Post-TAVR-Related Morbidity and Mortality: A Meta-Analysis. JACC Cardiovasc. Imaging.

[46] He X., Li Y., Wang Y., Tian W., Li Z., Ge L., Wang G., Chen Z. (2024). Prognostic Value of CT-Derived Myocardial Biomarkers: Extracellular Volume Fraction and Strain in Patients with Severe Aortic Stenosis Undergoing Transcatheter Aortic Valve Replacement: A Systematic Review and Meta-analysis. Acad. Radiol..

[47] Canessa M., Thamman R., Americo C., Soca G., Dayan V. (2021). Global Longitudinal Strain Predicts Survival and Left Ventricular Function After Mitral Valve Surgery: A Meta-analysis. Semin. Thorac. Cardiovasc. Surg..

[48] Kaur S., Jain V., Sadana D., Gillinov A.M., Desai M.Y., Griffin B.P., Xu B. (2020). Prognostic Utility of Left Ventricular Global Longitudinal Strain in Surgery for Primary Mitral Regurgitation: A Systematic Review. JACC Cardiovasc. Imaging.

[49] Farsalinos K.E., Daraban A.M., Ünlü S., Thomas J.D., Badano L.P., Voigt J.-U. (2015). Head-to-head comparison of global longitudinal strain measurements among nine different vendors: The EACVI/ASE inter-vendor comparison study. J. Am. Soc. Echocardiogr..

[50] Rösner A., Barbosa D., Aarsæther E., Kjønås D., Schirmer H., D’hooge J. (2015). The influence of frame rate on two-dimensional speckle-tracking strain measurements: A study on silico-simulated models and images recorded in patients. Eur. Heart J. Cardiovasc. Imaging.

[51] Sonaglioni A., Nicolosi G.L., Granato A., Bonanomi A., Rigamonti E., Lombardo M. (2024). Influence of chest wall conformation on reproducibility of main echocardiographic indices of left ventricular systolic function. Minerva Cardiol. Angiol..

[52] Nielsen M.Ø., Ljoki A., Zerahn B., Jensen L.T., Kristensen B. (2024). Reproducibility and Repeatability in Focus: Evaluating LVEF Measurements with 3D Echocardiography by Medical Technologists. Diagnostics.

